# Quadricuspid aortic valve repair: Results of a phenotype-based approach

**DOI:** 10.1016/j.xjtc.2025.01.009

**Published:** 2025-01-23

**Authors:** Karen B. Abeln, Jan M. Federspiel, Lennart Froede, Hans-Joachim Schäfers

**Affiliations:** a Department of Cardiovascular Surgery, Saarland University Medical Center and Saarland University, Homburg/Saar, Germany; b Department of Legal Medicine, Faculty of Medicine, Saarland University, Homburg/Saar, Germany; c Department of Cardiac Surgery, University Hospital Quirónsalud Madrid, Madrid, Spain

**Keywords:** aortic valve regurgitation, aortic valve repair, quadricuspid aortic valve

## Abstract

**Objective:**

Quadricuspid aortic valve (QAV) anatomy is a rare congenital anomaly. Patients develop relevant aortic regurgitation (AR), commonly between the fourth and sixth decades of life. Various approaches to repair have been proposed, but mid-term data are lacking. The present study aimed to investigate late results of QAV repair using different repair concepts.

**Methods:**

Between 2004 and 2023, 19 patients (32% male; mean age, 46 ± 12 years; range, 26-60 years) underwent QAV repair. The mean duration of follow-up was 6.3 ± 5 years (range, 4 months to 19 years), and follow-up was 96% complete. Patients underwent surgery for isolated AR (n = 18) or combined valve disease (n = 1). Three patients (16%) had concomitant ascending aortic dilatation.

**Results:**

The majority of patients underwent design change—tricuspidization (n = 13; 68%) or bicuspidization (n = 3; 16%)—of the QAV; the valve was left quadricuspid in 3 patients (16%). Sinotubular junction remodeling was performed by adding a sinotubular suture (n = 7; 37%) or by ascending aortic replacement (n = 3; 16%). All patients were alive at 5 years and 10 years postoperation; 2 required late aortic valve reoperation. Freedom from reoperation was 82% at 12 years (86% after tricuspidizdation and 67% after bicuspidization). The 3 patients whose valve remained quadricuspid did not require reoperation after 2 years, 3 years, and 5 years. Overall freedom from AR >2 was 76% at 12 years. At last follow-up, 2 patients had a mean gradient of 21 mm Hg, both of whom had undergone bicuspidization.

**Conclusions:**

QAVs can be repaired by different methods, including tricuspidization and bicuspidization. If the quadricuspid morphology is preserved, stabilization of annular and sinotubular dimensions may achieve a stable result.

**Figure:**
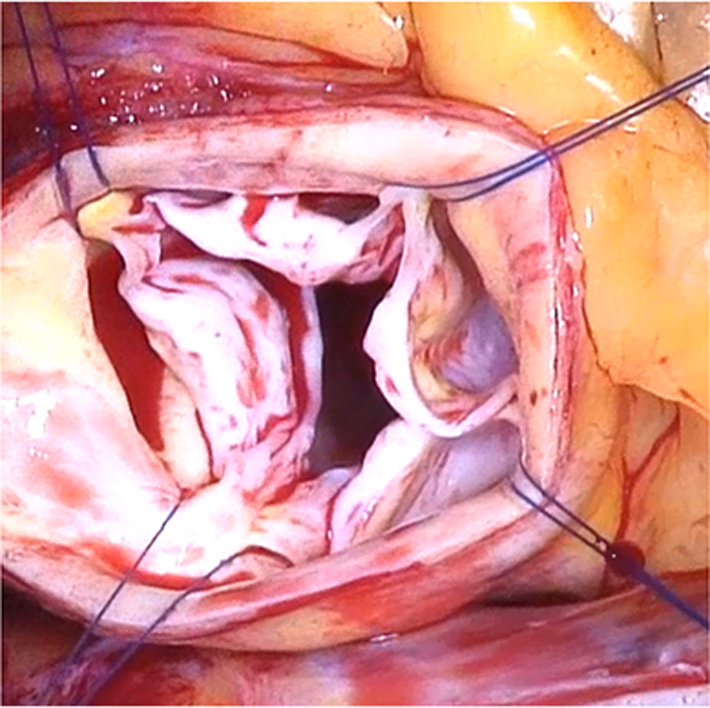
Intraoperative image of a quadricuspid aortic valve

**Central Message**

A regurgitant quadricuspid aortic valve can be treated by reducing root dimensions and by tricuspidization or bicuspidization if guided by repair concepts and valve symmetry.

**Perspective**

Experience with quadricuspid aortic valve (QAV) repair is limited. Coaptation in the QAV can be improved by reducing annular and particularly sinotubular junction dimensions; tricuspidization and bicuspidization are additional concepts. All these approaches may lead to good durability if adequate valve coaptation is achieved and the valve has a normal form.

Quadricuspid aortic valve (QAV) is frequent in truncus arteriosus but very rare in a normally developed aorta; different phenotypes have been described.^1^ Patients with QAV commonly develop aortic regurgitation (AR) in the fourth to sixth decade of life^2^ and thus require surgery at a relatively young age. The choice of procedure is difficult; commonly the valve is replaced using conventional valve substitutes.^3^ In recent years, the shortcomings of prosthetic valve replacement in younger adults,^3^^,^^4^ as well as the limitations of homografts for aortic valve replacement,^5^ have led to a renewed interest in valve repair.

Repair appears as an ideal option especially if the mechanism of AR in QAV can be corrected reproducibly. Echocardiographically, AR in QAV is characterized by a central coaptation defect that is most likely related to cusp restriction associated with the additional commissure, thus limiting cusp adaptation. Alternatively, dilatation of the sinotubular junction (STJ) may be involved in the mechanism of AR.^6^^,^^7^ In addition, the central cusp margins are commonly thickened and fibrotic, limiting cusp coaptation.

Various repair approaches have been proposed for a QAV or truncus valve.^7^, ^8^, ^9^, ^10^, ^11^ The precise repair approach for truncus valves has not always been described in detail; however, in infancy, better results were obtained when the quadricuspid design was changed into a tricuspid or bicuspid design.^12^ Nonetheless, freedom from reoperation after such repairs was only 55.0 ± 10.4% at 5 years.^12^

Based on the echocardiographic observations in QAV, repair should aim at improving central coaptation of the cusps. Treatment of the fibrotic thickening can involve thinning of the central free cusp margins. If STJ dilatation is present, reducing its size seems to be a logical maneuver. Design conversion to tricuspid or bicuspid configuration may be an additional tool. A design change from quadricuspid to tricuspid was proposed by us^7^ and others^9^ to address the restriction from the additional commissure. Others have proposed the creation of a bicuspid design by detaching 2 commissures.^8^

To date, reports on QAV repair are scarce and limited to case studies and small series of patients,^13^ generally with short follow-up. Based on our early experience,^7^ we have repaired all QAVs whenever encountered. The purpose of this study was to assess mid-term results of isolated QAV repair, comparing the different repair concepts.

## Patients and Methods

### Patients

We conducted a retrospective analysis of patients who underwent aortic valve surgery for a regurgitant QAV at Saarland University Medical Center between December 2004 and April 2023. Pure stenosis was not encountered. Combined aortic valve disease was defined as mean aortic valve gradient ≥20 mm Hg and AR grade ≥2.^14^ The primary endpoints were survival and freedom from reoperation; late AR was a secondary endpoint.

### Ethics Statement

The investigation was approved by the Saarland Regional Ethics Committee (CEP 203/19; approved May 2019). Individual patient consent was waived for the analysis and publication of data in an anonymized fashion.

### Surgical Technique

Preoperatively, aortic size was determined by transesophageal echocardiography, with a particular focus on annular and STJ diameter (Video 1). The chest was opened by median sternotomy, and the patient was connected to cardiopulmonary bypass using aortic and right atrial cannulation. The aorta was opened by transverse aortotomy approximately 10 mm above the STJ. Blood cardioplegia was provided directly into the coronary ostia. For assessment, stay sutures were placed in each commissure and kept under tension, maintaining the circumferential orientation of the commissures.

The valve was assessed systematically, paying attention to the quality of cusp tissue, cusp mobility, and presence and extent of fibrosis of the free cusp margins. In addition, the circumferential orientation of the commissures was noted, differentiating between symmetric and asymmetric types.^1^ Cusp stay sutures were placed in the corresponding parts of the free cusp margins, ensuring that equidistant parts were aligned, as measured from the corresponding commissure. Cusp size and configuration were then assessed by measuring geometric height^15^ and effective height.^16^ Prolapse was defined as an effective height of <9 mm or a free margin at least 2 mm lower than the remaining margins. An annuloplasty was added for annular size ≥24 mm^17^; it was placed prior to the cusp repair. For STJ diameter >27 mm to 30 mm, a decision for STJ size reduction was made by adding a Dacron graft or a sinotubular PTFE suture. The PTFE suture was placed before closing the aorta and left untied at that time. It was tied later under echocardiographic control on the beating heart.

Thickened free cusp margins were thinned. Redundant cusp tissue in the fused cusp (ie, prolapse) was corrected by central 5-0 polypropylene sutures on the free margin. Thus, the neocusps are brought to an identical level with the other cusps until the effective height was 9 mm and all free margins were at an identical level.

**Figure 1:**
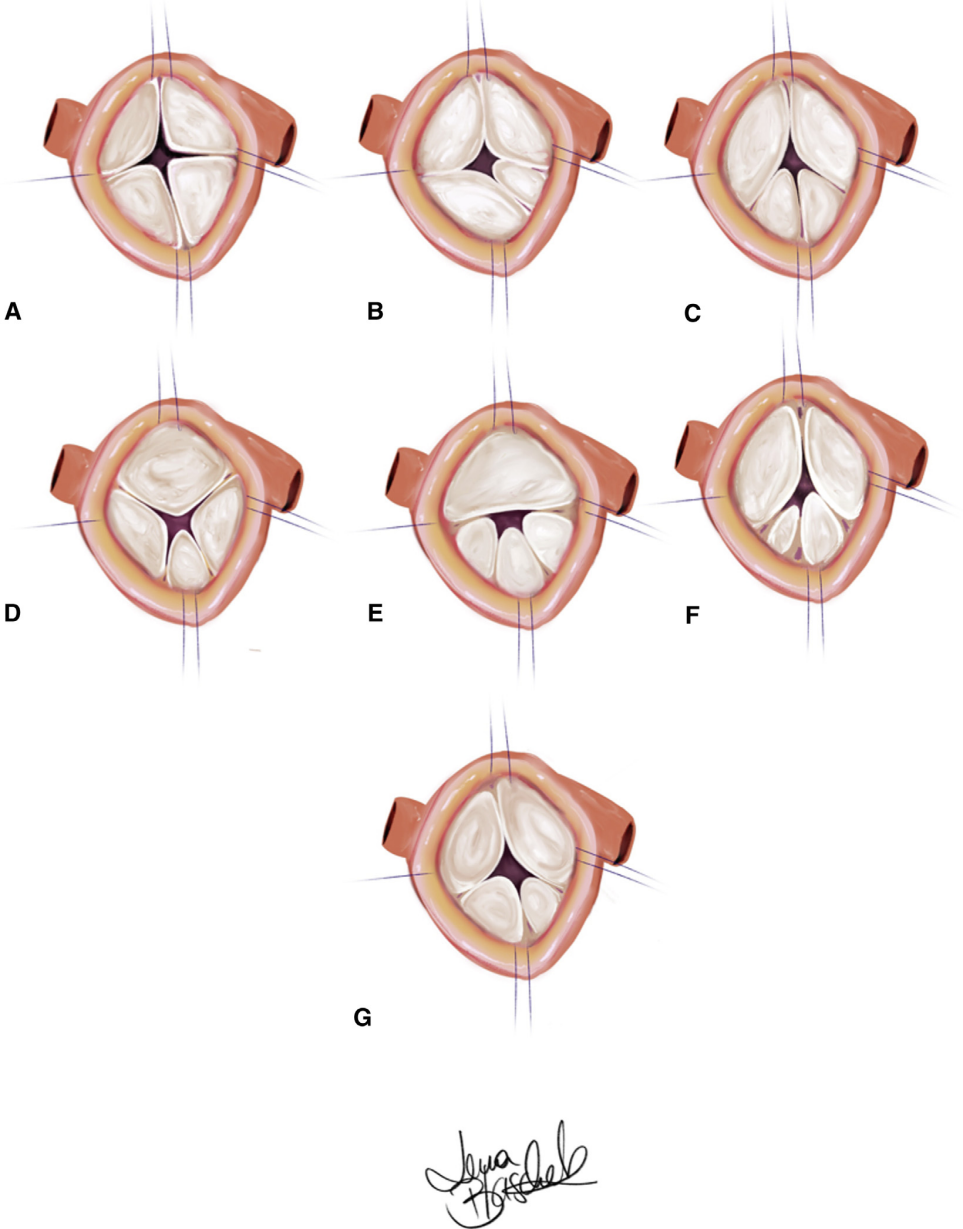
Anatomic description of the different types of quadricuspid aortic valves according to the Hurwitz classification.^1^ A, Symmetric valve with 4 equal-sized cusps. B, Asymmetric valve with 3 equal-sized cusps and 1 smaller cusp. C, Asymmetric valve with 2 equal-sized larger cusps and 2 equal-sized smaller cusps. D, Asymmetric valve with 1 large cusp, 2 mid-sized cusps, and 1 smaller cusp. E, Asymmetric valve with 1 large cusp and 3 equal-sized smaller cusps. F, Asymmetric valve with 2 equal-sized larger cusps and 2 unequal-sized smaller cusps. G, Asymmetric valve with 4 unequal-sized cusps.

**Figure 2:**
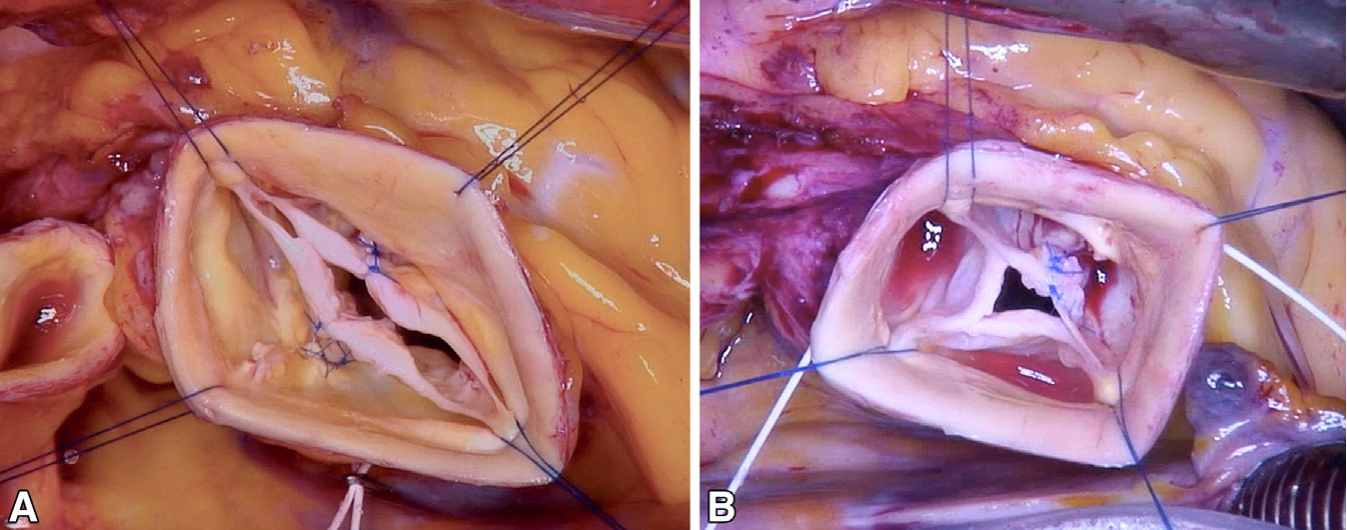
A, Intraoperative image of a bicuspidized quadricuspid aortic valve (QAV). B, Intraoperative image of a tricuspidized QAV.

In general, the symmetry of commissural distribution was taken into consideration in the decision between no design change or design change (ie, tricuspidization and bicuspidization). If the commissural and sinus distribution was symmetric (Hurwitz type A; Figure 1)^1^—that is, 4 cusps and sinuses of similar size—an attempt was made to improve central coaptation by reducing the STJ and, if necessary, the annulus, along with thinning the central cusp margins. If this did not result in good coaptation, the valve design was changed into a bicuspid configuration. Two opposing commissures were detached, and the respective cusps were adapted to create 2 joint cusps (Figure 2, *A*; Video 1).

With asymmetric configuration of the QAV (Hurwitz type B-G; Figure 1)^1^—that is, 2 cusps and sinuses smaller than the others—the valve was converted to a tricuspid design^18^ (Figure 2, *B*). The commissure between the 2 smaller cusps was detached, and the 2 adjacent cusps were adapted using single polypropylene sutures. A small pericardial patch was added if the tissue appeared restrictive on directly adapting the 2 cusps. Central cusp plication suture was added to correct any residual prolapse. Cusp configuration was adjusted to achieve an effective height of 9 mm.

Following aortic closure and deairing, transesophageal echocardiography was performed to assess valve function. If residual AR >1 was observed and a sinotubular suture had been placed, it was gently tightened under echocardiographic control and tied until AR was ≤1.

### Follow-up

All patients were followed prospectively, both clinically and echocardiographically (at discharge, 3 months, 1 year, and yearly thereafter). The patients were seen by their referring cardiologist or in our clinic. Echocardiograms from our institution and referring cardiologists were reviewed. Systolic gradients were measured using continuous-wave Doppler. AR was determined using color Doppler according to European guidelines.^14^

The mean and median follow-up were 6. 3 ± 5 years and 4 years (range, 4 months to 19 years). Follow-up was 96% complete (119 patient-years).

### Statistical Analysis

Statistical analyses were performed using SPSS 28.0 (IBM). Categorical variables are expressed as frequency (%). Non-normally distributed variables are presented as median and interquartile range; continuous variables, as mean ± standard deviation. Time-dependent data were assessed using the Kaplan-Meier method and the log-rank test. Survival and freedom from reoperation were calculated at 1, 5, 10, and 12 years. All tests were 2-sided, and a *P* value < .05 was considered statistically significant for all analyses.

## Results

### Patients

Overall, 19 patients with a QAV underwent surgery between December 2004 and April 2023; all were repaired and included in the present study. The cohort was 28% male and ranged in age from 26 to 60 years; the mean age was 46 ± 12 years (Table 1). The primary indication for surgery was relevant and symptomatic AR in all patients. In 18 patients, AR was present with (n = 10) or without (n = 8) root dilatation. One patient had combined aortic valve dysfunction. Concomitant ascending aortic aneurysm was present in 1 patient and in combination with root dilatation in 3 patients. Echocardiographically, all patients had a visible central coaptation defect with a central regurgitant jet. Thirteen patients were classified as Hurwitz type B, C, D, E, F, or G, and 6 patients were classified as Hurwitz type A (Figure 1).

**Table 1:** Patient characteristics

Patient	Sex	Age, y	Hurwitz classification	Annulus, mm	Sinus of Valsalva, mm	STJ, mm	Indication	LVEF <50%	LV dilatation	Prior aortic valve operation
1	F	46	B	23	32	25	AR	-	Yes	-
2	M	45	F	22	30	25	AR	-	Yes	-
3	F	45	C	23	32	25	AR	-	Yes	-
4	M	46	F	24	28	25	AR	-	-	-
5	F	60	G	26	33	32	AR	-	-	-
6	F	45	B	26	25	32	AR/AS	-	Yes	-
7	F	44	G	20	32	25	AR	-	Yes	-
8	M	48	D	30	34	25	AR	Yes	Yes	-
9	F	32	B	28	33	26	AR	Yes	Yes	-
10	M	26	A	30	42	32	AR	-	-	Yes∗
11	M	57	A	25	33	28	AR	-	-	-
12	F	55	E	27	33	30	AR	-	Yes	-
13	F	27	a	29	40	30	AR	Yes	Yes	Yes†
14	F	58	f	22	34	30	AR	-	-	-
15	F	50	b	25	31	30	AR	-	-	-
16	F	43	a	26	41	32	AR	-	-	-
17	M	49	a	24	33	26	AR	-	Yes	-
18	F	54	c	24	33	30	AR	-	Yes	-
19	F	54	a	24	26	28	AR	-	Yes	-

*STJ*, Sinotubular junction; *LVEF*, left ventricular ejection fraction; *LV*, left ventricle; *AR*, aortic regurgitation; *AS*, aortic stenosis.

∗ Pulmonary autograft replacement with a quadricuspid autograft.

† Truncus arteriosus repair.

Two patients had undergone previous cardiac surgery, including repair of truncus arteriosus in 1 patients and a Ross procedure using a quadricuspid pulmonary autograft in the other patient. Both of these patients underwent concomitant replacement of the degenerated right ventricular conduit.

### Early

Early findings included cusp prolapse in 7 patients (37%), calcifications in 1 patient (5%), cusp restriction in 9 patients (47%), and perforations in 2 patients (11%). The majority of patients underwent conversion to a tricuspid design (n = 13; 58%) or bicuspid design (n = 3; 26%). In the remaining 3 patients (n = 3; 16%), the valve was left quadricuspid. A suture annuloplasty was added in 7 patients (37%). STJ remodeling was performed by adding a sinotubular suture (n = 7; 37%) or by ascending aortic replacement (n = 4; 21%).

Ascending aortic replacement was performed as root remodeling (n = 3) or as tubular ascending aortic replacement (n = 1). Mean myocardial ischemia time ranged from 21 to 87 minutes; extracorporeal circulation time ranged from 31 to 144 minutes (mean, 38 ± 18 minutes) (Table 2).

**Table 2:** Perioperative data

Patient	gH, mm	eH, mm	Root remodeling	Sinotubular remodeling	Repair	Annuloplasty, mm	Patch∗	Follow-up, y	Reoperation	AR grade	Mean gradient	Last AR grade	Last mean gradient
1	20	9	-	-	Tri	-	Yes	14.2	-	2	5	1	6
2	20	8	-	-	Tri	-	Yes	1.3	-	1	5	1	6
3	20	4	-	-	Tri	-	Yes	12.5	-	1	4	1	6
4	20	9	-	-	Tri	-	Yes	10.2	-	1	5	2	8
5	18	9	-	Yes	Tri	23	-	10	-	0	5	1	10
6	20	9	-	Yes	Tri	23	-	13.1	-	1-2	5	2	8
7	21	10	-	-	Tri	-	-	14.4	-	1	6	2	8
8	22	11	-	-	Tri	25	-	9.3	-	1-2	6	1	7
9	20	9	-	Yes†	Tri	23	-	3.7	Yes	4	3	1	3
10	20	9	Yes	-	Qua	25	-	5.2	-	0-1	2	0	4
11	19	9	-	-	Tri	-	-	5	-	2	4	1	4
12	20	10	-	Yes	Qua	25	-	3.3	-	1	7	2	6
13	20	8	Yes	Yes†	Qua	25	-	2.1	-	0-1	2	1	5
14	19	9	-	Yes	Tri	-	-	3	-	0-1	10	1	12
15	19	9	-	Yes	Tri	-	-	2	-	0-1	8	1	3
16	19	9	Yes	Yes†	Bic	23	-	0.4	Yes	4	21	1	4
17	19	9	-	-	Bic	-	-	0.9	-	0	5	1	23
18	20	10	-	Yes	Tri	-	-	1	-	0-1	5	1	4
19	20	10	-	Yes	Bic	-	-	0.1	-	0	10	0	12

*gH*, Geometric height; *eH*, effective height; *AR*, aortic regurgitation; *Tri*, tricuspidization; *Bic*, bicuspidization; *Qua*, kept quadricuspid.

∗ Performed with autologous pericardium.

† Performed as ascending aortic replacement.

There were no early deaths, myocardial infarctions, or neurologic complications. No patient required a permanent pacemaker implantation. There were no early reoperations. Seventeen patients had AR ≤1 at discharge, and 2 patients had AR 2, both of whom had undergone tricuspidization. The mean systolic gradient at discharge was 6.5 ± 4.9 mm Hg (range, 3-21 mm Hg); it was 5.5 mm Hg after tricuspidization, 12 mm Hg after bicuspidization, and 3 mm Hg after no design change.

### Late

**Figure 3:**
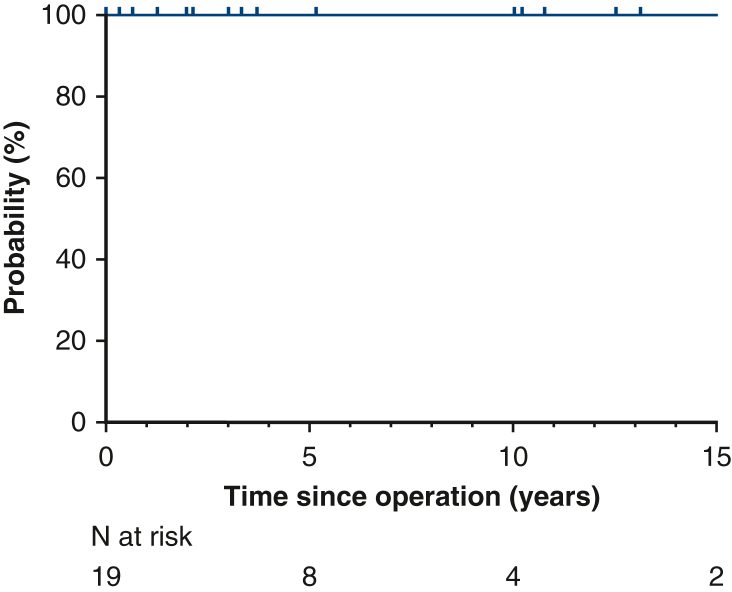
Survival (95% confidence interval).

**Figure 4:**
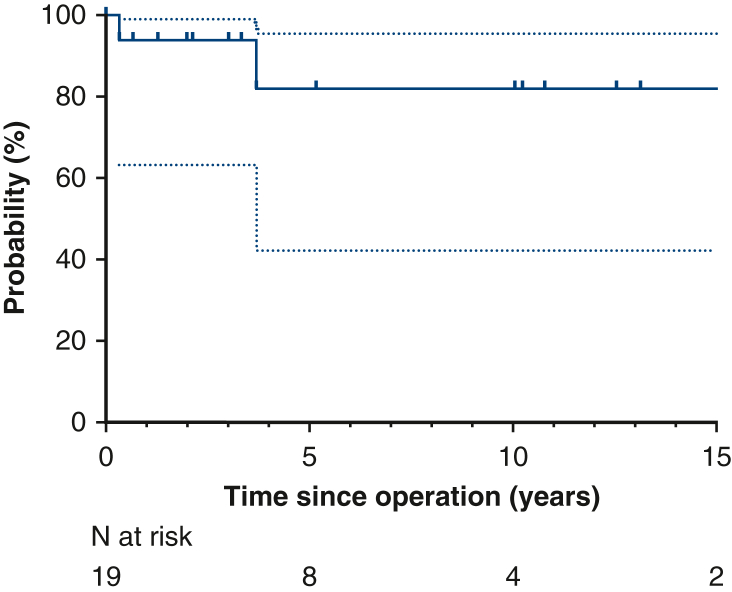
Freedom from aortic valve reoperation (95% confidence interval).

There were no late deaths. Survival was 100% at 5 years and 10 years (Figure 3). Freedom from reoperation at 12 years was 82% overall, 86% after tricuspidizdation, and 67% after bicuspidization (Figure 4). The 3 patients whose valve remained quadricuspid did not require reoperation after 2 years, 3 years, and 5 years, respectively.

**Figure 5:**
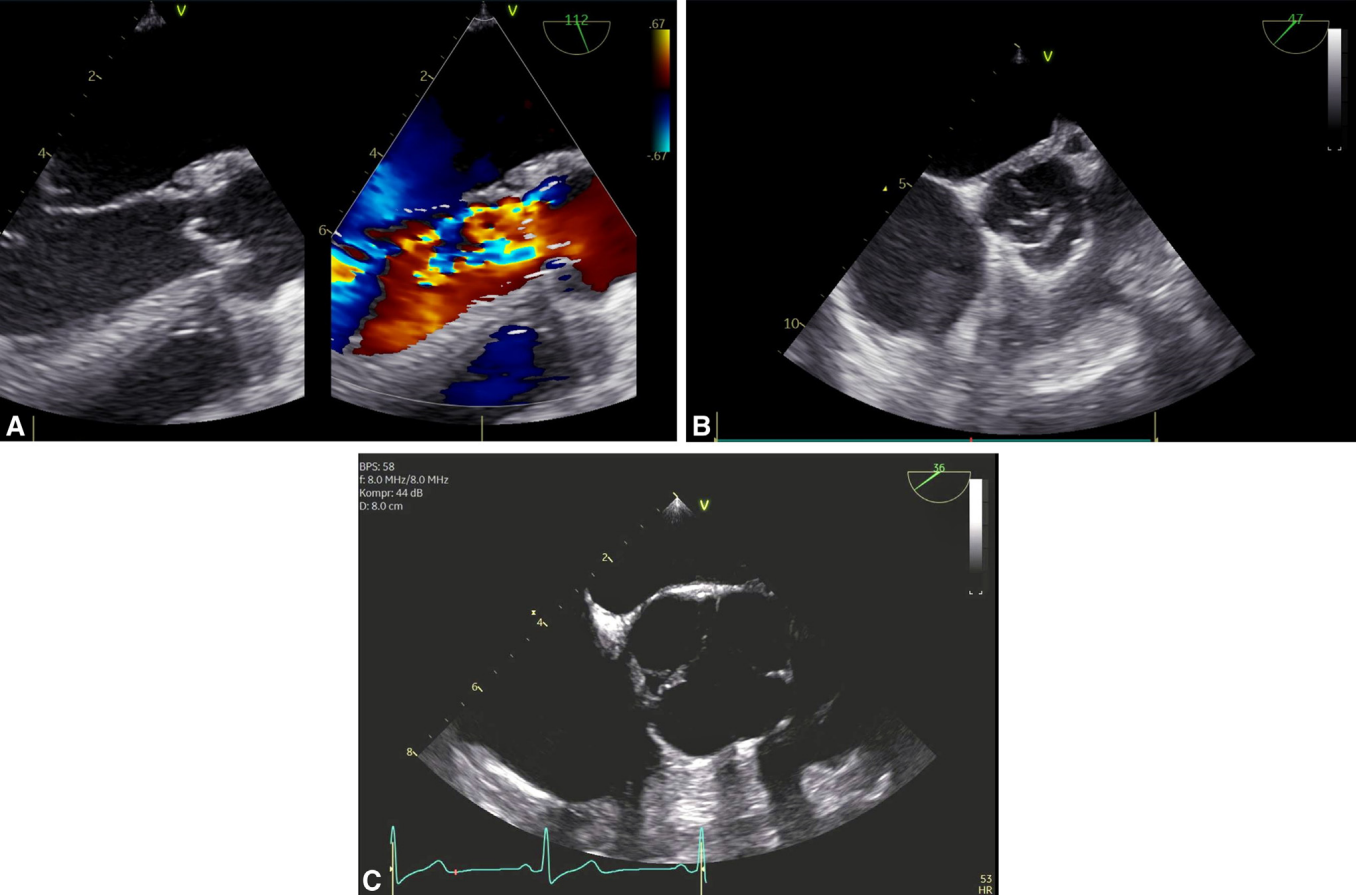
A, Intraoperative transesophageal long-axis view showing a larger conjoint new sinus compared to the other 2 sinuses with an asymmetric configuration after failed quadricuspid aortic valve (QAV) tricuspidization and severe aortic regurgitation. B, Intraoperative transesophageal short-axis view showing a larger conjoint new sinus compared to the other 2 sinuses with an asymmetric configuration after failed QAV tricuspidization. C, Intraoperative transesophageal short-axis view showing some asymmetry after failed QAV bicuspidization.

There were 2 late reoperations. One patient initially underwent tricuspidization of a type B QAV, combined with suture annuloplasty and ascending aortic replacement. Reoperation was necessary after 3.6 years for recurrent AR (Figure 5, *A*). At reoperation, prolapse of the newly created cusp was present. The new (ie, conjoint) sinus was larger than the other 2 (Figure 5, *A* and *B*), deviating from a symmetric tricuspid configuration. The valve was replaced with a mechanical prosthesis.

The second patient underwent bicuspidization, ascending aortic replacement (40 mm), and suture annuloplasty. She developed recurrent AR at 6 months postoperatively and was reoperated after 8 months. At reoperation, the adapting sutures of the 2 parts of one cusp were found to be torn, while the cusp tissue itself appeared preserved. In addition, the sinus circumference appeared larger than 50%, resulting in some asymmetry (Figure 5, *C*). We re-repaired the valve by resuturing the 2 parts of the respective cusps and reduced the sinus circumference by plication. At the last follow-up (1.5 years postoperatively), the valve remained competent with no trace of AR. The mean gradient was 3 mm Hg, and the root diameters were within normal range (annulus, 20 mm; sinus, 32 mm).

### AR

Of the 17 patients who did not require a reoperation, 13 (76%) had no or only trivial AR grade 1 at last follow-up. Three patients (17%) had AR grade 2 (tricuspidized, n = 2; kept QAV, n = 1), and 1 patient had AR grade 3 (tricuspidized, n = 1) at last follow-up.

**Figure E1:**
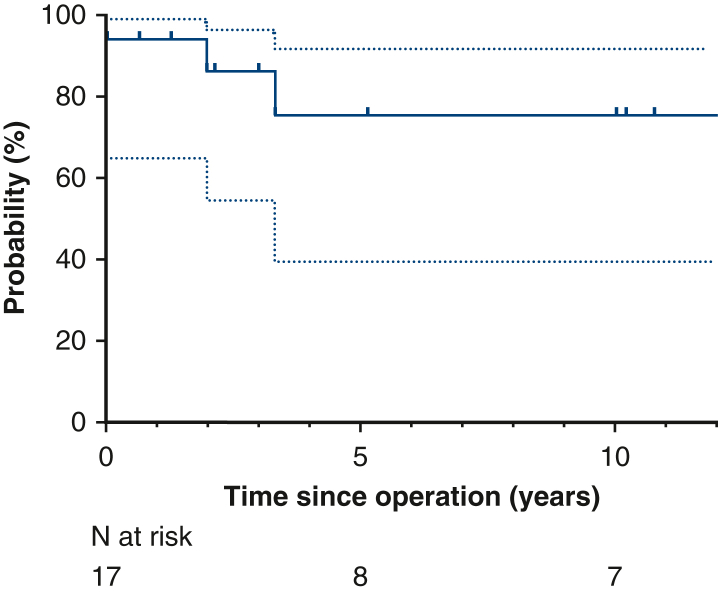
Freedom from aortic regurgitation >2 at last follow-up (excluding reoperations) (95% confidence interval).

Freedom from AR >2 at 12 years was 76% overall, 33% after bicuspidization, and 86% after tricuspidization (*P* = .008). Excluding reoperated patients, freedom from AR ≥2 at 12 years was 75% overall, 50% after bicuspidization, 50% after no design change (ie, configuration remained quadricuspid), and 90% after tricuspidization (*P* = .388) (Figure E1).

### Gradients

At last follow-up, the mean gradient was 10 ± 4 mm Hg overall, 4 ± 2 mm Hg without a design change, 6.4 ± 3 mm Hg after tricuspidization, and 16 ± 4 mm Hg after bicuspidization (Figure E1). Two of the patients who underwent bicuspidization had a mean gradient of 23 mm Hg.

## Discussion

QAV morphology is a rare cause of AR, and the affected patients usually become symptomatic at a relatively young age.^19^ Given the shortcomings of conventional aortic valve replacement,^3^^,^^4^ repair leading to superior results regarding valve-related complications, and possibly survival, should be desirable.^7^^,^^20^ This requires that the mechanism of AR can be addressed reliably with such an approach.

Valve repair has been performed in QAVs of truncus arteriosus.^7^, ^8^, ^9^, ^10^, ^11^ These procedures are commonly performed, but difficult, in infants with root dilatation. After all, any repair must allow for somatic growth, and thus permanent stabilization of root dimensions is impossible. QAVs in adulthood pose a different scenario, in which root dimensions can be reduced and stabilized permanently.

Echocardiographically, AR in QAV is consistently associated with a central coaptation defect. The most likely causes of this cusp restriction are related to the additional commissure and possibly also to STJ dilatation. Another pathologic feature inhibiting coaptation may be the thickened and fibrotic substance of the central cusp margins. This raises the question of whether coaptation can be achieved by reduction of the STJ alone or whether additional maneuvers are necessary. Shaving of the cusp margins or changing the valve configuration may principally improve cusp coaptation. Another approach is reducing the number of commissures, thereby relieving cusp restriction and improving coaptation.

Previous procedures have involved a design change, that is, conversion of the valve into a tricuspid or bicuspid design through detachment of 1 or 2 commissures.^7^^,^^8^ Both approaches have been used, and available data on mid-term repair durability are limited. In fact, data are confined to case reports and small series of patients.^7^, ^8^, ^9^, ^10^^,^^12^^,^^13^ In 1 larger cohort, most valves were replaced.^13^

Initially, we felt that a successful repair should involve a design modification of the valve by reducing the number of commissures to 3, because shaving the central cusp margins did not result in adequate coaptation. This was supported by the coincidence that the first QAVs encountered in our series were asymmetric according to the Hurwitz classification.^1^ Thus, when repairing these valves we created tricuspid configurations, using the established concepts of geometric^15^ and effective height.^16^ With this approach, lack of cusp tissue may necessitate the insertion of a limited pericardial patch, which has a good prognosis in tricuspid aortic valve repair.^21^

Although the results were satisfactory, we soon realized that this approach might not be ideal for the symmetric Hurwitz type A, to which we applied the concept of bicuspidization.^8^ Finally, because not all valves exhibited marked fibrosis of the central cusp margins, we decided to reduce annular and STJ dimensions if cusp tissue appeared to be preserved.

Our results show that the apparent mechanism of AR—predominantly cusp restriction— could be relieved by all 3 approaches. The best results and longest follow-up were achieved with tricuspidization, even though the difference between the tricuspid and bicuspid designs was not significant. In addition, the reduction and stabilization of annular and STJ dimensions achieved the functional goal of reducing or eliminating AR. Conceptually, creation of a bicuspid configuration may be easier in terms of surgical judgment of cusp coaptation. Nonetheless, both involve suturing cusp tissue, which may be prone to subsequent suture disruption.^22^ At present, it is too early to determine whether perfect (tricuspid or bicuspid) valve symmetry is important for long-term repair durability, as has been found in bicuspid aortic valve repair.^23^ This would be supported by re-repair in bicuspidized QAV in which the cusp tissue was resutured and the symmetry was improved, leading to a good, stable result. While increased gradients were observed in conjunction with bicuspidization of QAV, these gradients were not relevant, and both concepts appear to be valid. It seems logical that the symmetry of the Hurwitz types should be taken into consideration when choosing one approach over the other.

It may be speculated that more QAVs can be preserved and repaired by simply improving coaptation through reduction of the annulus and STJ.^24^ This would involve the least degree of cusp maneuvers and possibly facilitate surgical judgment of adequate valve configuration. If marked asymmetry or more pronounced cusp alterations are encountered, replacement of the aortic valve, including the use of a pulmonary autograft, should be considered.

### Limitations

The main limitation of this study is its observational design. Although data of consecutive procedures were obtained prospectively, the analysis was performed retrospectively, and treatment allocation was not randomized. The reproducibility of our findings may be limited due to the institutional conditions, that is, experienced surgeons performing the procedures in a high-volume center. Finally, QAVs have a wide range of phenotypes with differing pathologic alterations. Although the main concepts have worked in our experience, the precise choice of technical details will depend on these features; the overall limited experience does not allow for generalization. Despite these limitations, to our knowledge this study is one of few studies investigating the durability of repair of isolated QAVs in adults.

## Conclusions

In conclusion, AR in the presence of QAV can be treated by reducing annular and STJ dimensions. In addition, tricuspidization or bicuspidization of a QAV are reproducible repair techniques if adequate valve configuration is achieved and the technique is guided by the symmetry of the valve. In selected cases, the valve may be kept quadricuspid if the concepts of aortic valve repair are applied.

## Conflict of Interest Statement

The authors reported no conflicts of interest.

The *Journal* policy requires editors and reviewers to disclose conflicts of interest and to decline handling or reviewing manuscripts for which they may have a conflict of interest. The editors and reviewers of this article have no conflicts of interest.

## Supplementary Data

Video 1 Echocardiographic description and detailed surgical steps of a quadricuspid aortic valve bicuspidization.Video available at: https://www.jtcvs.org/article/S2666-2507(25)00044-6/fulltext.
