# Supplementary figures and images for: Quadricuspid aortic valve repair: Results of a phenotype-based approach
Source: JTCVS Tech. 2025 Jan 23;30:23–31. doi: 10.1016/j.xjtc.2025.01.009 (PMC11998587; doi:10.1016/j.xjtc.2025.01.009)

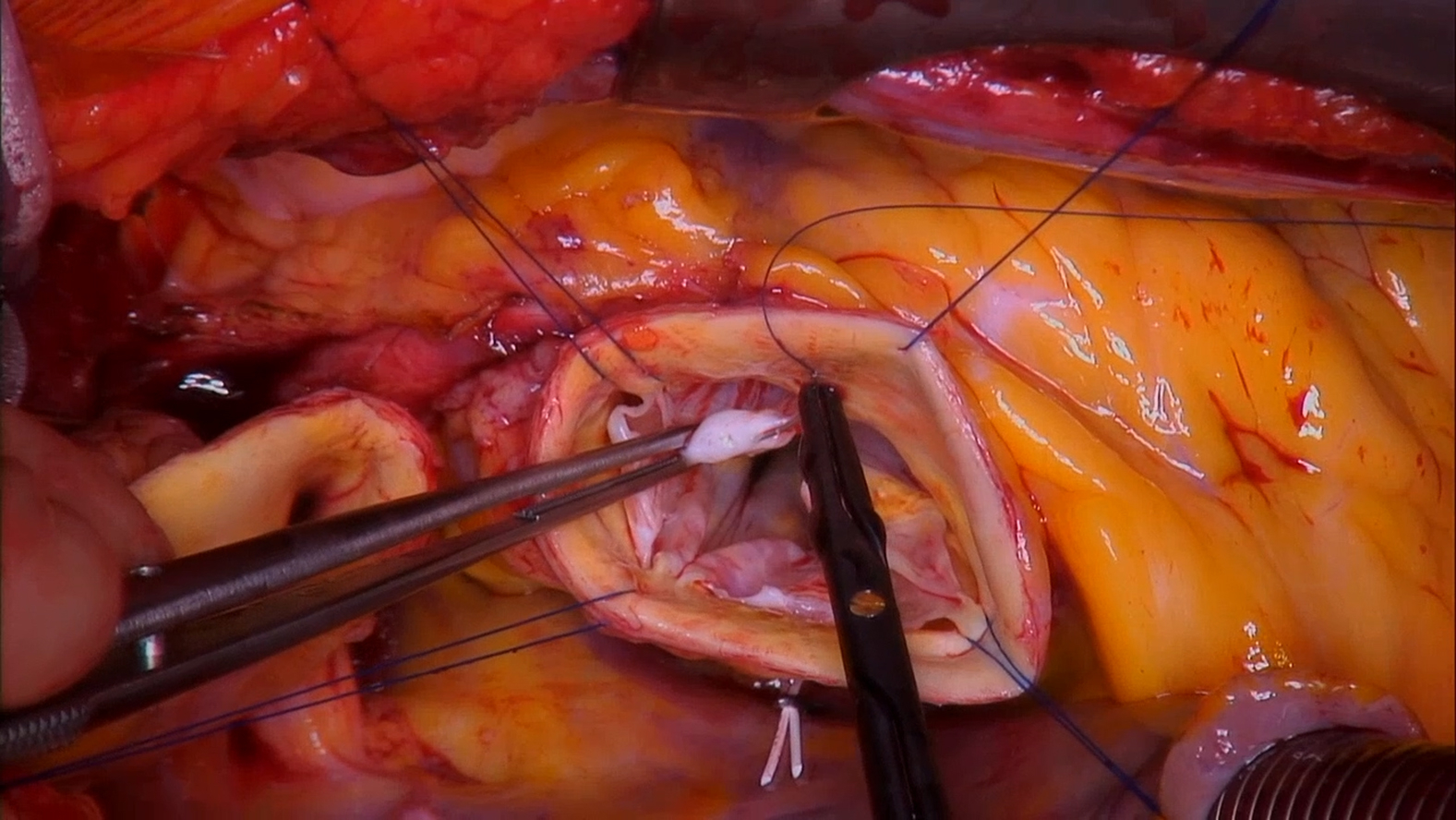

Supplement: Video 1 — Echocardiographic description and detailed surgical steps of a quadricuspid aortic valve bicuspidization.Video available at: https://www.jtcvs.org/article/S2666-2507(25)00044-6/fulltext. [file fx2.jpg]
